# Current Modulation of Guanylate Cyclase Pathway Activity—Mechanism and Clinical Implications

**DOI:** 10.3390/molecules26113418

**Published:** 2021-06-04

**Authors:** Grzegorz Grześk, Alicja Nowaczyk

**Affiliations:** 1 Department of Cardiology and Clinical Pharmacology, Faculty of Health Sciences, Ludwik Rydygier Collegium Medicum in Bydgoszcz, Nicolaus Copernicus University in Toruń, 75 Ujejskiego St., 85-168 Bydgoszcz, Poland; g.grzesk@cm.umk.pl; 2 Department of Organic Chemistry, Faculty of Pharmacy, Ludwik Rydygier Collegium Medicum in Bydgoszcz, Nicolaus Copernicus University in Toruń, 2 dr. A. Jurasza St., 85-094 Bydgoszcz, Poland

**Keywords:** guanylate cyclase (GC), chronic heart failure (CHF), pulmonary arterial hypertension (PAH)

## Abstract

For years, guanylate cyclase seemed to be homogenic and tissue nonspecific enzyme; however, in the last few years, in light of preclinical and clinical trials, it became an interesting target for pharmacological intervention. There are several possible options leading to an increase in cyclic guanosine monophosphate concentrations. The first one is related to the uses of analogues of natriuretic peptides. The second is related to increasing levels of natriuretic peptides by the inhibition of degradation. The third leads to an increase in cyclic guanosine monophosphate concentration by the inhibition of its degradation by the inhibition of phosphodiesterase type 5. The last option involves increasing the concentration of cyclic guanosine monophosphate by the additional direct activation of soluble guanylate cyclase. Treatment based on the modulation of guanylate cyclase function is one of the most promising technologies in pharmacology. Pharmacological intervention is stable, effective and safe. Especially interesting is the role of stimulators and activators of soluble guanylate cyclase, which are able to increase the enzymatic activity to generate cyclic guanosine monophosphate independently of nitric oxide. Moreover, most of these agents are effective in chronic treatment in heart failure patients and pulmonary hypertension, and have potential to be a first line option.

## 1. Introduction

Since the beginning of the 21st century, the treatment of metabolic disturbances-related diseases of the cardiovascular system has become deeper. Previously, the target of treatment was rather clinical, whereas now, the clinical answer is important but the tools have changed; now, they are generally metabolic. For years, GCs had seemed to be an homogenic and tissue nonspecific enzyme, whereas within the last few years, in light of preclinical and clinical trials, it became an interesting an target for pharmacological intervention. This review will focus on the role of direct and indirect modulation of guanylate cyclases in cardiovascular pharmacology.

## 2. Guanylate Cyclases

In mammals, there are two key types of guanylate cyclases (GC), classified according to localization of enzymes in the cell. The first is called guanylate cyclase-coupled receptor or membrane-bound guanylate cyclase (mGC). The second is completely intracellular, soluble (sGC). Agonists for mGC are peptides, such as natriuretic peptides type A, B, C, whereas for sGC, they are gaseous mediators, such as nitric oxide and carbon monoxide [1].

In detail, there are four soluble guanylate cyclase subunits, marked α1, α2, β1 and β2, and transmembrane forms named with consecutive letters of the alphabet, from A to G (Table 1). Dimer is a typical and minimal form of catalytic unit [2,3]. Transmembrane forms exist as homodimers, whereas soluble forms are heterodimers. One of the mGCs, type C, seems to be different—homotrimer [2,3,4,5].

**Figure 1:**
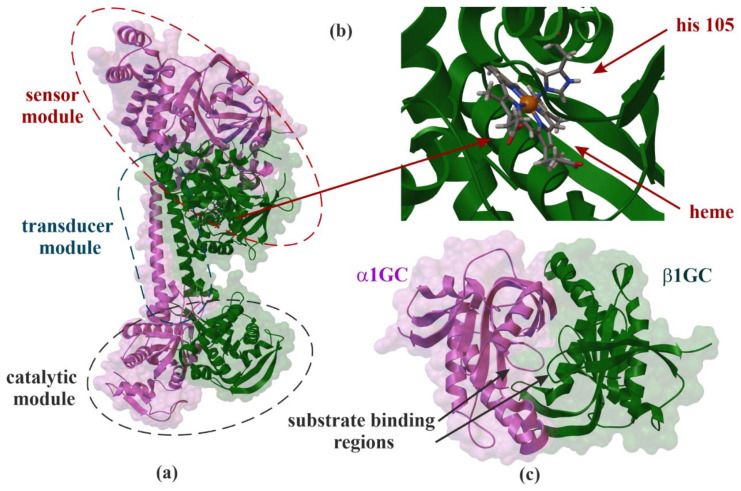
X-ray structure of soluble guanylate cyclase (sGC). Domain organization of the human α1β1GC heterodimer (PDB ID: 6JT1, 3.9 Å) (**a**) [16], interaction side view between heme and histidine 105 (his 105) (**b**) and α1β1GC catalytic domains that resembles the Chinese yin-yang symbol with both subunits arranged in a head-to-tail conformation (PDB ID: 4NI2, 1.9 Å) (**c**) [17].

Soluble GCs (Figure 1, EC 4.6.1.2) were found in many different tissues, especially brain, cardiovascular system, kidney and lungs [5]. All four units—α1, α2, β1 and β2—were identified in humans. At the amino acid level, the α1 subunit found in humans is about 34% identical to the β1 subunit, whereas the α2 subunit is 48% homologous to the α1 subunit [6,7,8]. Experiments performed on mice suggest that α1 is the major subunit in platelets and lungs. Meanwhile, α2 subunits are responsible for about 6% of activity of sGCs in vasculature, but in response to NO in mice, lacking α1 is sufficient to achieve maximal vascular smooth muscle relaxation [8]. Further, β1 contains several interesting binding domains, such as about 200 residues in the aminoterminal heme prosthetic group and about 250 residues of carboxyl-terminal GC domain. In this condition, compared to the β1 isoform, β2 subunit has an additional 86 carboxyl-terminal amino acids sequence for isoprenylation or carboxymethylation [9]. The relation between sCG and hemoglobin is interesting. The β1 subunit is the axial ligand of the pentacoordinated reduced iron center of heme. The binding place, His-105, is located at the amino terminus of the β1 subunit (Figure 1). This kind of binding is necessary for both NO- and CO-dependent activation. NO binds to the heme ring at sixth position. It breaks the bond between the iron and axial histidine to form a five-coordinated ring with NO in the fifth position. sGC can be activated by other gaseous mediator CO, but it binds heme to form a six-coordinated complex [10]. This interaction can partially explain the hyperreactivity of vascular smooth muscle after blood transfusions. Disruption of β1 subunit leads to significant reduction in NO-dependent vascular relaxation and platelet aggregation, whereas disruption of α1 subunit leads to loss of platelet aggregation only. The male mice lacking β1 subunit are infertile. Homozygous knockout animals presented gastrointestinal obstruction similar to the cGMP dependent protein kinase G (PKG). Molecular studies suggest that sGC is one of the most important enzymes in the cardiovascular system and targets for possible pharmacological intervention. Increased vascular resistance in cases with reduced NO bioavailability secondary to the endothelial damage can be effectively bypassed by direct intervention at the sGC level. Mergia et al. [8] confirmed that GC activation is sufficient to mediate vasorelaxation not only at higher NO concentrations, but by direct enzyme stimulation. The results of study presented by Wedel et al. suggested that mutation of six conserved histidine residues reduced—but did not abolish—nitric oxide stimulation, whereas a change of His-105 to phenylalanine in the beta 1 subunit yielded a heterodimer that retained basal cyclase activity but failed to respond to nitric oxide supporting thesis that direct activation can be more effective option than heme-related intervention [10]. The results presented by Friebe et al. confirmed the crucial role of sGC as a receptor for NO in different smooth muscle reactivity related settings [11]. The loss of cGMP-dependent protein kinase I abolishes nitric oxide dependent relaxation of smooth muscle, resulting in severe vascular and intestinal dysfunctions confirming role of sGC pathway in relaxation of smooth muscle [12], especially signalling pathway mediated by IP3 receptor associated cGMP-dependent protein kinase type I substrate which is highly expressed in smooth muscle of cardiovascular and gastrointestinal tissue is essential for smooth muscle relaxation by NO/cGMP and ANP/cGMP [13]. The GC pathway, especially sGC, is crucial enzyme mediating relaxation of different types of smooth muscles [14,15]; thus, it is a very interesting target for achieving therapeutic goals, especially in diseases secondary to atherosclerosis process with common endothelial dysfunction and decreased NO bioavailability.

Guanylate cyclase-coupled receptors (mGC) are activated by peptides such as atrial natriuretic peptides (NP). The first type of mGC is called GC-A and is activated by NP type A (ANP) and B (BNP). The lack of ANP stimulation leads to cardiovascular hypertrophy with hypertensive vessel response, whereas isolated BNP deficiency is a situation leading to hypertrophy without hyperreactivity of vascular smooth muscle cells [18,19,20,21]. GC type B (GC-B) is activated by NP type C (CNP). Structurally, it is similar to the GC-A, but predominantly localized in bone and ovary tissue make the key actions in stimulation of endochondral ossification required for long bone growth and oocyte maturation. Because of its localization in the cardiovascular system, smooth muscle cells and fibroblasts GC-B seem to be one of the most important mGCs in heart failure [18,22,23,24,25,26,27,28]. GC type C is activated by different peptide agonists: guanylin and uroguanylin. GC-C present on apical membrane of epithelial cells is a target for heat stable enterotoxins leading by elevation of intracellular cGMP concentration and PKG-dependent phosphorylation of the cystic fibrosis transmembrane regulator to increased Cl^−^ secretion in gut [29]. An animal model of mice lacking functional GC-C resistance to heat stable endotoxin (Salmonella enterica serovar Typhimurium) was found [30]. Inhibition of guanylin stimulation increased colonic epithelial cell proliferation with no influence on vessel smooth muscle cells contractility and sodium excretion. In this condition, uroguanylin signaling inhibition decreased the ability to excrete an enteral sodium and chlorides ions load and induced salt-independent hypertension [31,32]. The influence on the process of epithelial cells proliferation seems to be a possible therapeutic target, i.e., in the treatment of colon cancer [29,33,34]. Type D of GC was found in the olfactory system. GC-D can be activated by peptides guanylin and uroguanylin, but not heat stable enterotoxins and additionally by carbon dioxide and bicarbonate. In mice, GC-D participates in food preference response [35,36,37]. GCs type E and F are involved in the process of producing vision. Both types are expressed in retina and GC-E additionally in pineal gland. Inactivation of enzymes leads to rod and cone dystrophy and blindness [38,39]. GC-G was found in rat small intestine. The structure is similar to NP receptors, but elevation of activity of GC was not dependent on ANP, BNP, CNP and heat stable enterotoxins. Some authors suggest bicarbonate as an activator [40]. In mice, GC-G serves as an unusual receptor in Grueneberg ganglion of the anterior nasal region neurons mediating the detection of the volatile alarm pheromones especially substance 2-sec-butyl-4,5-dihydrothiazole [41,42].

Animal studies suggested the significant role of modulation of GC function in pathogenesis of diseases especially of the cardiovascular system, intestinal, skeletal, visual system and fertility. There are some possibilities of pharmacological intervention. An increase in the activity of GC may be achieved directly or indirectly. Direct pharmacological intervention is the simplest way to modify receptor or enzyme answers. sGCs can be activated by NO or NO donors such as sodium nitroprusside; thus, modulation of vascular smooth muscle reactivity was used in different clinical settings such as arterial hypertension and pulmonary arterial hypertension (PAH) to achieve rapid blood pressure stabilization in heart failure patients. This pathway of the modulation of vessel function was effective for different contraction models [43,44,45] and different clinical settings [46,47,48,49,50]. In this condition, the source of NO can be nitric oxide produced by nitric oxide synthase or drug and, thus, exogenous NO or NO-donor.

The key molecule in this system is cGMP. The produced cGMP is responsible for numerous effects in cells. Most of them are guided by PKG pathway. Activation of PKG is responsible for the activation of myosin phosphatase, which, in turn, leads to the release of calcium from intracellular stores in smooth muscle cells, finally leading to vascular smooth muscle relaxation. Phosphorylation by the PKG of vasodilator-stimulated phosphoprotein (VASP) is responsible for the decrease in platelet activation level and the activation of a number of transcription factors which can lead to changes in gene expression, which, in turn, can alter the response of the cell to a variety of stimuli [12,13,14,15].

## 3. NO Production

Nitric oxide synthase is an enzyme that catalyzes the synthesis of nitric oxide in two different steps. The first one is the oxidation of L-arginine to Nω-hydroxy-L-arginine; then, the substrate under the influence of NOS and oxygen is decomposed into L-citrulline, accompanied by the release of nitric oxide from vascular endothelial cells. Three basic types of nitric oxide synthase have been distinguished, now called NOS-1, NOS-2 and NOS-3. In the recently used nomenclature, they were designated NOS-1: nNOS (neuronal NOS), NOS-2: iNOS (inducible NOS) and NOS-3: eNOS (endothelial NOS), respectively. Type 1 synthase is localized primarily in the central and peripheral nervous system, skeletal muscles, pancreatic islets, endometrium and nephron dense macula. The basic physiological tasks of NOS-1 include modulating nerve transmission and regulating the function of the nephron or regulating intestinal peristalsis. Nitric oxide produced by NOS-1 can also act as a neurotransmitter, especially in the vegetative system known as non-adrenergic non-cholinergic (NANC). Type 2 synthase is located mainly in macrophages, cardiac striated muscle cells, liver, smooth muscle and vascular endothelium, and is synthesized as part of the response to infection, inflammation or sepsis, under the influence of inflammatory cytokines (mainly interleukin-1, interferon-γ or TNF-α). The activated enzyme remains active for several hours, synthesizing significant amounts of nitric oxide [51]. Nitric oxide produced by NOS-3 plays primarily a role as a regulator of muscle tone in the local vascular endothelial-vascular muscular system. It is also a factor that inhibits the adhesion and aggregation of platelets and angiogenesis. The role of NOS-3, as an element initiating the activation of NOS-2 under the influence of lipopolysaccharides, is emphasized. In experimental studies on the hyporeactivity of vessels treated with lipopolysaccharides, it was suggested that synthase located in the vascular endothelium was involved as the first link in the development of vascular hyporeactivity in sepsis [46]. In studies on isolated animal tissues exposed to short exposure to lipopolysaccharides, a statistically significant inhibition of NOS-2 expression was demonstrated in the case of a previous blockage of NOS-3 activity. Such results suggest that nitric oxide synthesized by NOS-3 may be a mediator of inflammation in sepsis [52] and confirmed results presented by Grześk in 2001 [46] and 2003 [48]. The results confirm the results of subsequent experiments, which showed that the lack of NOS-3 prevents from full expression of NOS-2 in the presence of lipopolysaccharides, which suggests that in the pathogenesis of sepsis there is primary activation of NOS-3, followed by the released nitric oxide being a pro-inflammatory stimulus for expression NOS-2 [53].

## 4. Pharmacological Intervention

**Figure 2:**
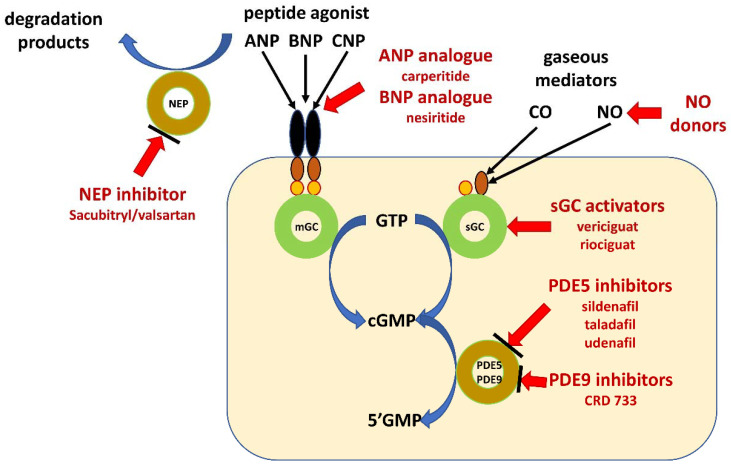
Schematic illustration of pharmacological intervention in guanylate cyclase/cyclic GMP pathway. Guanylate cyclase-coupled receptor, membrane-bound guanylyl cyclase (mGC); soluble guanylate cyclase (sGC); natriuretic peptides type A, B and C (ANP, BNP and CNP); neprilysin (NEP); gaseous mediators–nitric oxide (NO) and carbon monoxide (CO); cyclic guanosine monophosphate (cGMP); guanosine triphosphate (GTP); guanosine monophosphate (GMP); phosphodiesterase type x (PDEx).

There are several possible options leading to increase in cGMP concentrations. The first one is related to the use of analogues of ANP and BNP. The second is the increasing ANP and BNP by inhibition of degradation of peptides. The third one leads to increase in cGMP concentration by inhibition of its degradation by inhibition of phosphodiesterase type 5. The last option is the increasing concentration of cGMP by additional direct activation of sGC (Figure 2).

At the clinical level, pharmacological intervention seems to be dedicated for all clinical settings with low bioavailability of nitric oxide. In clinical medicine, there is a huge family of diseases with a vascular endothelium as one of the core symptoms. The depressed GC-pathway signaling is common in a family of atherosclerosis related diseases such as coronary artery disease, heart failure and hypertension, but also diabetes and hypercholesterolemia. It explains why side effects are very rare during the stimulation of this pathway, but, if are they present, are related to the overstimulation in non-target tissues. The overproduction of NO is seen especially in acute diseases such as septic shock. The common side effects are low blood pressure, blurred vision, confusion, dizziness, faintness or lightheadedness when getting up suddenly from a lying or sitting position, pale skin, sweating, trouble breathing, unusual bleeding or bruising, unusual tiredness or weakness. On the other hand, the predominant therapeutic effect for all studies and all therapeutic pathways is the same: to decrease mortality, hospitalization rate and increase quality of life.

### 4.1. Natriuretic Peptides Analogues

According to the current guidelines, pharmacological intervention with NO or NO-donors as stimulators of sGC are considered only for short-time actions, whereas in chronic treatment, these are not recommended [54,55,56]. What is interesting is the possibility of activation of mGC, located in cells of the cardiovascular system; thus, types activated by NPs type A, B and C GC-A and GC-B can be stimulated by physiological agonists, ANP, BNP and CNP, but with the analogs too. The first attempt at this kind of intervention was made after the discovery of analogues of NPs: carperitide, analogue of ANP and nesiritide, an analogue of BNP. Carperitide is a recombinant α-human atrial natriuretic peptide, leading to vasodilation. It is indicated for the treatment of patients with acute heart failure (including acute exacerbation of chronic heart failure). Moreover, carperitide was approved by the Pharmaceuticals and Medical Devices Agency of Japan (PMDA). Nesiritide is the recombinant form of BNP. Nesiritide works to facilitate cardiovascular fluid homeostasis through the counterregulation of the renin–angiotensin–aldosterone system, stimulating cGMP, leading to smooth muscle cell relaxation. Both mechanisms were important in the prevention of progression in the exacerbation of heart failure and chronic kidney disease. Nesiritide was investigated as a pharmacological agent indicated in acute decompensated congestive heart failure. It was registered in the United States Food and Drug Administration (FDA) for this purpose in 2001 after initial non-approval. Mitaka et al. [57] analyzed the risk of cardiovascular and renal effects of carperitide and nesiritide for preventing and treating acute kidney injury in cardiovascular surgery patients. A meta-analysis showed that carperitide infusion significantly decreased peak serum creatinine levels, incidence of arrhythmia and renal replacement therapy. The meta-analysis also showed that carperitide or nesiritide infusion significantly decreased the length of intensive care unit stay and hospital stay vs. controls. The authors concluded that NP treatment is an interesting option to preserve postoperative renal function in cardiovascular surgery patients [57]. Sezai et al. [58] investigated the efficacy of carperitide treatment for high-risk patients undergoing coronary artery bypass grafting (CABG). In a randomized controlled trial of 367 high-risk patients undergoing CABG, the primary endpoint was major adverse cardiovascular and cerebrovascular events. There was no significant difference in survival between the carpetide and placebo groups (*p* = 0.1651), but no patient from the carperitide group started hemodialysis after operation, but 7 patients did in the placebo group and the dialysis rate was significantly lower in the carperitide group (*p* = 0.0147). Serum creatinine and BNP were also significantly lower in the carperitide group at 1 year postoperatively. The authors concluded that in the early postoperative period, carperitide has a cardiorenal protective effect that prevents postoperative cardiovascular and cerebrovascular events and hemodialysis. Perioperative low-dose carperitide infusion was found useful in high-risk patients undergoing on-pump CABG [58]. Zhao et al. [59] prepared a meta-analysis of the efficacy and safety of nesiritide in patients with acute myocardial infarction and heart failure. The results of trials involving 870 participants were included in the meta-analysis. Nesiritide treatment significantly increased left ventricular ejection fraction, cardiac index and 24 and 72 h urine volumes. Additionally, pulmonary capillary wedge pressure, right atrial pressure and BNP and N-terminal brain natriuretic peptide (NT-proBNP) levels were significantly decreased in patients treated with nesiritide, compared with those treated with control drugs (“control drugs” were optimal pharmacotherapy according to guidelines). The authors concluded that nesiritide appeared to improve cardiac function and, moreover, was safe for patients [59]. The results of large clinical trials presented by O’Conor et al. in 2011 failed to show a difference between nesiritide and placebo on mortality or rehospitalization rate in this group of patients [60]. Other studies suggest that protective effect is non-significant or borderline [61]; thus, large placebo-controlled studies must be performed to clarify the role of these agents in clinical medicine.

Therapeutic stimulation with analogues of NP is interesting, but, unfortunately, in all performed studies, populations were rather small and not homogenous. The multifactor etiology of heart failure can explain why the presented results were different. All the studies confirmed the safety of that therapeutic option and its efficacy in laboratory and echocardiographic parameters describing disease progress, highly suggesting that this therapeutic option must be considered. To clearly describe the parameters such as mortality and hospitalizations rate, large placebo-controlled studies must be performed to describe the role of NPs analogues in the treatment of heart failure.

### 4.2. Inhibition of Neprilysin

The second therapeutic option is the inhibition of degradation of NPs into inactive metabolites; thus, real tissue concentration becomes higher. Neprilysin (NEP, EC 3.4.24.11) is key enzyme responsible for degradation of vasoactive peptides, such as ANP, BNP and CNP, but also adrenomedullin, angiotensin I and II, bradykinin and vasoactive intestinal peptide. Some of these peptides, i.e., NPs or bradykinin, are responsible for vascular tone regulation and modulation of remodeling in cardiovascular system, especially in heart failure. The spectrum of NEP actions is wider and includes peptides involved in neurodegenerative diseases (i.e., amyloid β, neurotensin), inflammation processes (i.e., neurokinin A, calcitonin gene-related peptide), mitomitogenesis, angiogenesis and hypothalamic-pituary axis. McMurray et al. [62] published results of the PARADIGM-HF trial. The study drug, sacubitril/valsartan, was compared to the standard according to the current guidelines of therapy, including enalapril and angiotensin converting enzyme inhibitor. The study was a prospective, randomized, double-blind trial of 9,419 patients with NYHA class II-IV, heart failure and reduced left ventricular ejection fraction, with confirmation by elevated NP levels. The key exclusion criteria included symptomatic hypotension, SBP < 100 mm Hg, serum potassium >5.2 mmol/L, eGFR < 30 mL/min or a history of angioedema. The trial concluded early after meeting a pre-specified stopping point for compelling clinical benefit. After a median follow-up of 27 months, the sacubitril/valsartan group of patients had a 20% reduction in the combined endpoint of cardiovascular death or HF hospitalization. All-cause mortality was also significantly lower in the valsartan/sacubitril group (17% vs. 19.8%) [62]. Clinical beneficial effect is predominantly dependent on ANP increase and partially BNP, whereas there are no changes in CNP levels [63,64]. Meta-analysis from clinical trials suggest the presence of a beneficial effect only in patients with reduced EF, whereas in patients with EF > 45%, the effect is not significant [65,66]. Additional beneficial effects were present during dual path treatment including NEP inhibition and angiotensin converting enzyme [67]. The benefits of the treatment are partially dose-dependent. Patients with dose reduction effects were similar to the target dose group [68]. According to the current guidelines of the European Society of Cardiology, the top target is the treatment of heart failure with reduced ejection fraction patients. The spectrum of sacubitril action is much wider and includes the possibility of arterial hypertension treatment [69] and treatment of chronic kidney disease, especially in heart failure subjects [70]. Moreover, recently, a drug was approved by the FDA for the treatment of heart failure with preserved ejection fraction. In animal models, NEP plays a role in pathogenesis of several amyloid deposition diseases such as age-related macular degeneration, cerebral amyloid angiopathy or sensorimotor axonal polyneuropathy. As heart failure is a disease affecting elderly groups, neurological benefits are valuable [71,72]. Studies performed with NEP inhibitors clearly presented the relation between benefits and side effects. Pharmacological intervention was very effective and safe; thus, NEP inhibitors became one of key groups in the treatment of heart failure with a high possibility of an increase in their role in next guidelines.

Currently, NEP inhibition represents a powerful therapeutic tool in treating chronic heart failure, but data suggest a potential role for the use of that pathway in a broader spectrum of cardiovascular and non-cardiovascular disease.

### 4.3. Inhibition of Phosphodiesterases

In humans, three PDEs are selective and involved in cGMP actions. There are PDE5 located especially in cardiovascular system, PDE6 found in the retina and PDE9 present in heart muscle and brain. In this condition, modulation of PDE5 and PDE9 function is interesting as a cardiovascular therapeutic option [73,74,75].

The family of PDE5 inhibitors was discovered as a drug for heart failure treatment. The key metabolic path was related to an increase in cGMP concentration, as PDE5 is responsible for specific cGMP degradation. Currently, PDE5 inhibitors are an important therapeutic option in the treatment of pulmonary hypertension and their concentration is the highest in pulmonary circulation. PDE5 inhibitors are effective in the treatment of pulmonary arterial hypertension, both primary and associated with systemic connective tissue disease, in adults and children [76,77,78,79]. Pulmonary arterial hypertension is characterized by a reduction in the production of NO in the endothelium with a simultaneous increase in the expression and activity of PDE type 5 in smooth muscle cells of the pulmonary arteries [76]. In the SUPER study (Sildenafil Use in Pulmonary Arterial Hypertension), 12 week treatment with sildenafil in 278 patients with pulmonary arterial hypertension in WHO functional class II or III compared to placebo was associated with an extension of 6 min walk distance and a decrease in pulmonary vascular resistance [80]. Similar properties were also demonstrated by tadalafil, which was confirmed in the PHIRST (Pulmonary Arterial Hypertension and Response to Tadalafil) study [81]. According to the current guidelines of pharmacotherapy with PDE5 inhibitors is cornerstone of pulmonary hypertension therapy [77,78]. In heart failure with reduced ejection fraction, there are only some small clinical trials. A hemodynamic effect was confirmed. Sildenafil decreased both resting and stress pulmonary pressure, pulmonary resistance, increased cardiac index, right ventricle ejection fraction and improved peak oxygen consumption [82]. Unfortunately, PDE5 were not effective in the treatment of heart failure with preserved ejection fraction [83]. Some studies and case reports describe the effectiveness of PDE5 before and after heart transplant in patients with pulmonary hypertension secondary to left ventricle heart failure [84]. PDE5 inhibitors, by stimulating the GCs pathways, show a relatively selective vasodilator activity to the pulmonary arteries, and thus are one of the basic groups used in pharmacotherapy of pulmonary hypertension. Moreover, they can reverse their pathological remodeling and directly increase the contractility of the overloaded right ventricle. Although data from clinical trials are limited, it appears that these drugs may become an attractive and clinically beneficial therapeutic option for patients with heart failure and secondary pulmonary hypertension.

PDE9 inhibition increases cGMP signaling and attenuates stress-induced hypertrophic heart disease in preclinical studies. In the mouse transverse aortic constriction pressure overload heart failure model, a PDE9 inhibitor, CRD-733 treatment reversed existing LV hypertrophy, significantly improved left ventricle ejection fraction and attenuated left atrial dilation. CRD-733 prevented elevations in left ventricle end diastolic pressures [85]. These findings support future investigation into the therapeutic potential of CRD-733 in human heart failure [85,86,87]. Inhibition of brain PDE9 may improve synaptic plasticity, behavior; thus, PDE9 inhibitors have been advanced into initial clinical studies to assess the potential to improve cognitive function in patients with Alzheimer’s disease and schizophrenia [75,88].

The inhibition of PDE5 is commonly used as an element in multi-compound therapy of pulmonary hypertension and impotence, whereas historically, the primary target, treatment of heart failure due to side effects, failed because of low tissue selectivity in the cardiovascular system. In this condition, due to localization inside heart muscle cells and the brain, pharmacotherapy with novel PDE9 inhibitors seems to be very promising not only in cardiology, but in geriatrics too. According to the results of first studies, there is a huge chance for an effective and safe therapeutic option for both heart failure and dementia patients.

### 4.4. Direct Activation of Soluble Guanylate Cyclase

Modulators of sGC have been developed to target this important signaling cascade in the cardiovascular system. sGC stimulators display a dual directional action, synergistic effect with endogenous NO and direct stimulation of the native form of the enzyme independently of NO, resulting in increased cGMP production. sGC activators are able to activate the dysfunctional heme-free sGC, resulting in increased cGMP production even in reduced NO bioavailability [89,90]. In patients treated because of atherosclerosis dependent diseases such as heart failure, coronary artery disease, arterial hypertension and many others, the cornerstone of disease is endothelial dysfunction leading to depressed NO production; thus, heme-dependent activation of sGC and production of cGMP will be reduced. In that condition, there is only one possibility to stabilize cGMP levels; it is direct stimulation of sGC by its activators.

Riociguat is the first sGC stimulator to have made a successful transition from animal experiments to controlled clinical studies in patients [91]. After clinical evaluation, riociguat was accepted for the treatment of pulmonary arterial hypertension and chronic thromboembolic pulmonary hypertension. In pulmonary arterial hypertension patients, riociguat significantly improved exercise capacity and secondary efficacy endpoints in patients with pulmonary arterial hypertension. The 6 min walk distance had significantly increased by a mean of 30 m in the riociguat group and had decreased by a mean of 6 m in the placebo group. Riociguat improved the 6 min walk distance both in patients who were receiving no other treatment for the disease and in those who were receiving endothelin-receptor antagonists or prostanoids. There were significant hemodynamic improvements in pulmonary vascular resistance, NT-proBNP levels, WHO functional class, time to clinical worsening and Borg dyspnea score [92]. Patients with chronic thromboembolic pulmonary hypertension similar relations were observed in a riociguat group of patients: improvement in 6 min walk test, decrease in pulmonary vascular resistance, NT-proBNP levels, WHO functional class, quality of life [93]. Because of pharmacokinetic profile (short half lifetime) application of riociguat in other cardiovascular indications, such as heart failure, is limited [94].

Heart failure as clinical indication for vericiguat treatment was evaluated in clinical trials. One of the first of them was the SOCRATES-REDUCED study [95]. The key target was to determine the dosage and safety of vericiguat, a soluble guanylate cyclase stimulator, in patients with worsening chronic heart failure and reduced left ventricular ejection fraction. A total of 456 patients, clinically stable with ejection fraction less than 45% and a worsening chronic HF event. Vericiguat did not have a statistically significant effect on change in NT-proBNP level at 12 weeks but was well-tolerated. In the VICTORIA study, phase 3, randomized, double-blind, placebo-controlled trial, 5050 patients with chronic heart failure and an ejection fraction of less than 45% were enrolled. Patients were randomized to vericiguat or placebo, in addition to current guideline-based medical therapy. Among patients with high-risk heart failure, the incidence of death from cardiovascular causes or hospitalization for heart failure was lower among those who received vericiguat than among those who received placebo [96]. Moreover, results of studies in patients with chronic heart failure and preserved ejection fraction suggest that vericiguat treatment in current regimen is not effective, but continuation of large clinical trials is necessary [97,98]. Among patients with CHF with recent decompensation, a novel strategy of sGC activation was effective. Vericiguat compared with placebo was effective in reducing cardiovascular death or hospitalization for heart failure, but with no reduction in all-cause mortality. In this condition, vericiguat may represent a novel treatment option in heart failure patients with recent decompensation.

Clinical trial confirmed efficacy of small molecule sGC activator vericiguat in patients with heart failure with reduced ejection fraction. Due to the unequivocal results, it can be expected that this therapeutic option will be included in the nearest guidelines for the treatment of heart failure with reduced left ventricular systolic function. Unfortunately, treatment of heart failure with preserved systolic function has been difficult to date, as there is practically no therapeutic option with proven effectiveness. It is necessary to continue clinical trials conducted in relatively large and homogeneous populations in order to obtain conclusive results.

## 5. Conclusions and Perspective

Treatment based on modulation of GCs pathway function is one of the most promising technologies in pharmacology. Pharmacological intervention is not only effective and safe, but also provides a stable increase in the basal concentration of cGMP. The target for all therapeutic options is similar: to increase in cGMP level. The effect can be achieved by stimulation of guanylate cyclase-coupled receptors on cell surface with analogues of NPs, inhibition of degradation of cGMP with blockade of dependent enzymes PDE5 and PDE9 and inhibition of degradation of NPs into inactive metabolites by inhibition of neprilysin or direct stimulation of sGC. For all therapeutic options, benefits in laboratory and echocardiographic findings in cardiovascular diseases were confirmed.

Especially interesting is the role of sGC stimulators and sGC activators, able to increase the enzymatic activity of sGC to generate cGMP independently of NO. Moreover, most of these agents are effective in chronic treatment in heart failure patients and pulmonary hypertension and have a potential to be a first line option especially in patients with recurrent exacerbations. Additionally, the possibility to block PDE9 in heart failure and dementia patients is very interesting. The confirmation of mortality and hospitalization rate reduction was clearly presented for sGC activator, neprilysin inhibitor in heart failure patients and PDE5 inhibitors in pulmonary hypertension patients. For all groups of patients, it is necessary to continue clinical trials conducted in large and homogeneous populations in order to obtain conclusive results to modify and extend indications for current and novel agents.

The modulation of the GC pathway presents the potential to be a key therapeutic option in patients with atherosclerosis-related diseases, pulmonary hypertension and diseases with reduced cognitive function in elderlies.

## Figures and Tables

**Table 1:** Guanylyl cyclases: activators, tissue expression and physiological effects.

Guanylyl Cyclase	Tissue Expression	Physiological Activator	Key Effects
Soluble α1	Cardiovascular system, platelets, brain	NO, CO	Vasodilation, angiogenesis, inhibition of platelet aggregation
Soluble α2	Cardiovascular system, brain	NO, CO	Vasodilation, angiogenesis
Soluble β1	Cardiovascular system, platelets, brain	NO, CO	Vasodilation, angiogenesis inhibition of platelet aggregation, intestinal motility
Soluble β2	Gastrointestinal tract, liver, kidney	NO, CO	Apoptosis, inhibition of anti-apoptotic endothelin pathway
GC-A	Cardiovascular system (vascular smooth muscle, heart), lung, kidney, adrenal, adipose tissue	ANP, BNP	Vasodilation, angiogenesis, regulation of hypertrophy, remodeling processes
GC-B	Cardiovascular system (vascular smooth muscle, endothelium, heart), lung, bone, brain, liver, uterus, follicle	CNP	Vasodilation, angiogenesis. regulation of hypertrophy, remodeling processes, cartilage homeostasis and endochondral bone formation, regulation of female fertility
GC-C	Intestinal epithelium	Guanylin, uroguanylin and bacterial heat-stable enterotoxin	Regulation of colonic epithelial cell proliferation
GC-D	Olfactory bulb	Guanylin, uroguanylin, CO_2_/HCO_3_	guanylin- and uroguanylin-dependent olfactory signaling, food and odor preference response (mices)
GC-E	Retina, pineal gland	guanylyl cyclase activator proteins	Vision process
GC-F	Retina	guanylyl cyclase activator proteins	Vision process
GC-G	Olfactory bulb, lung, intestine, skeletal muscle, testes	Pheromones, CO_2_/HCO_3_	detection of the volatile alarm pheromones, kidney, ischemia/reperfusion preconditioning

## Data Availability

Not applicable.

## Abbreviation

cAMP	Cyclic Adenosine Monophosphate
CO	carbon monoxide
FDA	US Food and Drug Administration
GC	guanylate cyclase
GC-A	guanylate cyclase type A
GC-B	guanylate cyclase type B
GC-C	guanylate cyclase type C
GC-D	guanylate cyclase type D
GC-E	guanylate cyclase type E
GC-F	guanylate cyclase type F
GC-G	guanylate cyclase type G
sGC	soluble guanylate cyclase
mGC	guanylate cyclase-coupled receptor or membrane-bound guanylyl cyclase
GMP	guanosine monophosphate
cGMP	cyclic guanosine monophosphate
GTP	guanosine triphosphate
LPS	lipopolysaccharide/endotoxin
NP	natriuretic peptide
ANP	natriuretic peptide type A
BNP	natriuretic peptide type B
CNP	natriuretic peptide type C
NANC	non-adrenergic, non-cholinergic
NEP	neprilysin
NO	nitric oxide
NOS	nitric oxide synthase
NOS-1	neuronal nitric oxide synthase
NOS-2	cytokine-inducible nitric oxide synthase
NOS-3	endothelial nitric oxide synthase
PAH	pulmonary arterial hypertension
PDE5	Phosphodiesterase type 5
PDE9	Phosphodiesterase type 9
PKG	cGMP dependent protein kinase G
SNP	sodium nitroprusside
TNFα	tumor necrosis factor α
NT-proBNP	N-terminal pro-brain natriuretic peptide
VASP	vasodilator-stimulated phosphoprotein
